# Dupilumab for pediatric chronic cutaneous graft-versus-host disease with eczematous and sclerodermiform features^؆^

**DOI:** 10.1016/j.abd.2026.501496

**Published:** 2026-11-09

**Authors:** Fabià Torres-Betato, Helena Iznardo, Ivan López-Torija, Felipe Peirano, Luís Puig, Esther Roé

**Affiliations:** a Department of Dermatology, Hospital de la Santa Creu i Sant Pau, Sant Pau Research Institute, Universitat Autònoma de Barcelona, Barcelona, Spain; b Department of Pediatric Hematology and Oncology, Hospital de la Santa Creu i Sant Pau, Sant Pau Research Institute, Universitat Autònoma de Barcelona, Barcelona, Spain

Dear Editor,

Chronic cutaneous Graft-Versus-Host Disease (cGVHD) is a late complication of allogeneic hematopoietic stem cell transplantation characterized by inflammatory and fibrosing manifestations.^1^ Sclerodermiform cGVHD presents with progressive skin induration and fibrosis and is frequently refractory to conventional therapies. Although the pathogenesis of fibrotic cGVHD involves multiple immune pathways, including Th1, Th2-, and Th17-mediated responses together with profibrotic mediators such as TGF-β and PDGF,^2^ IL-4 and IL-13 have also been implicated in fibroblast activation and collagen deposition in experimental models and other fibrosing disorders,^3^ providing a potential rationale for IL-4/IL-13 blockade in selected patients. Dupilumab, a monoclonal antibody targeting the IL-4 receptor α subunit, has shown benefit in atopic dermatitis-like cGVHD, although evidence in paediatric sclerodermiform disease remains scarce.^4^

We report the case of a 13-year-old girl diagnosed with an autoimmune lymphoproliferative syndrome-like disorder who underwent HLA-identical allogeneic hematopoietic stem cell transplantation from bone marrow. The donor had no personal history of atopic disease. The post-transplant course was complicated by cytomegalovirus-associated secondary graft failure requiring two graded donor lymphocyte infusions.

Approximately 8-months after transplantation, the patient developed progressive xerosis, intense pruritus (peak itch-NRS 8/10), eczematous plaques involving 30% Body Surface Area (BSA), and diffuse skin induration involving the face and extremities, associated with functional limitation and reduced mobility. Physical examination revealed generalized eczematous dermatitis (EASI-23) with superimposed sclerodermiform thickening, particularly in the centrofacial region and distal extremities. Multiple preexisting keloidal scars on the hands and feet were also noted (Fig. 1). Skin biopsy showed spongiotic dermatitis with interface changes and superficial perivascular lymphocytic infiltrates compatible with cutaneous cGVHD with atopic dermatitis–like features. The biopsy was obtained from an eczematous lesion rather than from an indurated area. Given the characteristic clinical presentation, including progressive diffuse skin induration associated with functional limitation and reduced mobility, the diagnosis of slcerodermiform cutaneous cGVHD was established clinically according to the 2014 NIH Consensus Criteria for chronic GVHD.^1^ Following the onset of cGVHD, treatment with ruxolitinib 10 mg daily was initiated together with high-potency topical corticosteroids, topical tacrolimus, emollients, and systemic corticosteroids, achieving only partial and transient improvement. Despite approximately 12-months of treatment, the patient achieved only partial and transient improvement, with persistent severe pruritus, eczematous dermatitis, and functional limitation. Extracorporeal photopheresis was considered; however, given the marked eczematous phenotype and persistent pruritus, dupilumab was added to ongoing ruxolitinib therapy.

**Fig. 1:**
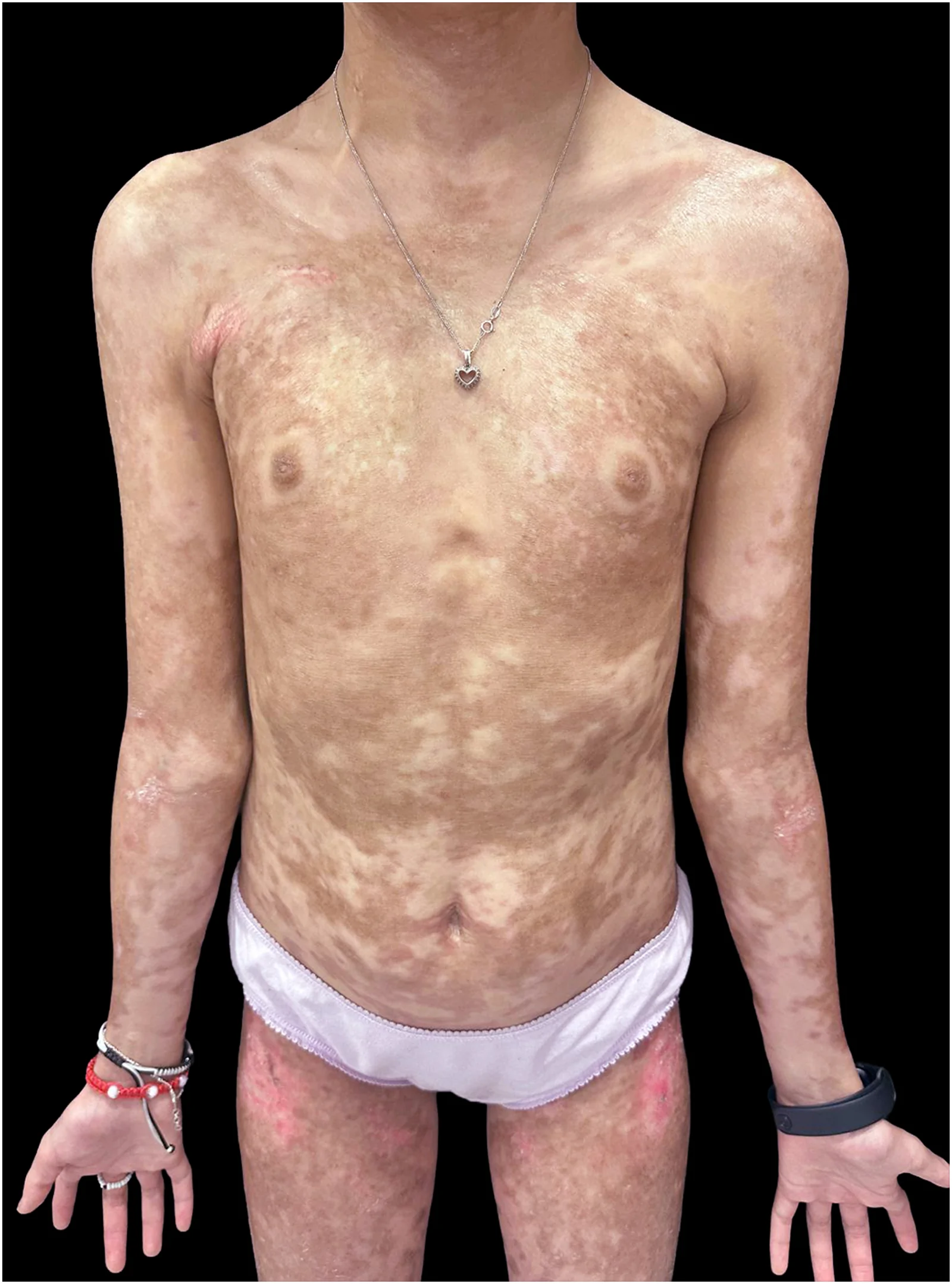
Diffuse cutaneous sclerosis with skin thickening involving the extremities and thorax prior to initiation of dupilumab.

Subcutaneous dupilumab was started with a loading dose of 400 mg followed by 200 mg every 2-weeks. After the initiation of dupilumab, the patient experienced rapid improvement in pruritus and eczematous lesions, with the itch-NRS decreasing from 8/10 to 5/10. At 6-weeks, the eczematous eruption had markedly improved (BSA 10%, EASI-8). Moreover, progressive softening of the skin induration and improvement in mobility were observed, especially in the face and extremities. In contrast, preexisting keloidal lesions became more prominent.

After 10-months of follow-up, the patient maintained sustained improvement in diffuse skin induration, pruritus (itch-NRS 2/10), and eczematous lesions (BSA 3%, EASI-2) (Fig. 2). Functional impairment decreased substantially, and no new areas of sclerosis appeared. Keloidal lesions remained enlarged but stabilized without further progression. No relevant adverse events were reported.

**Fig. 2:**
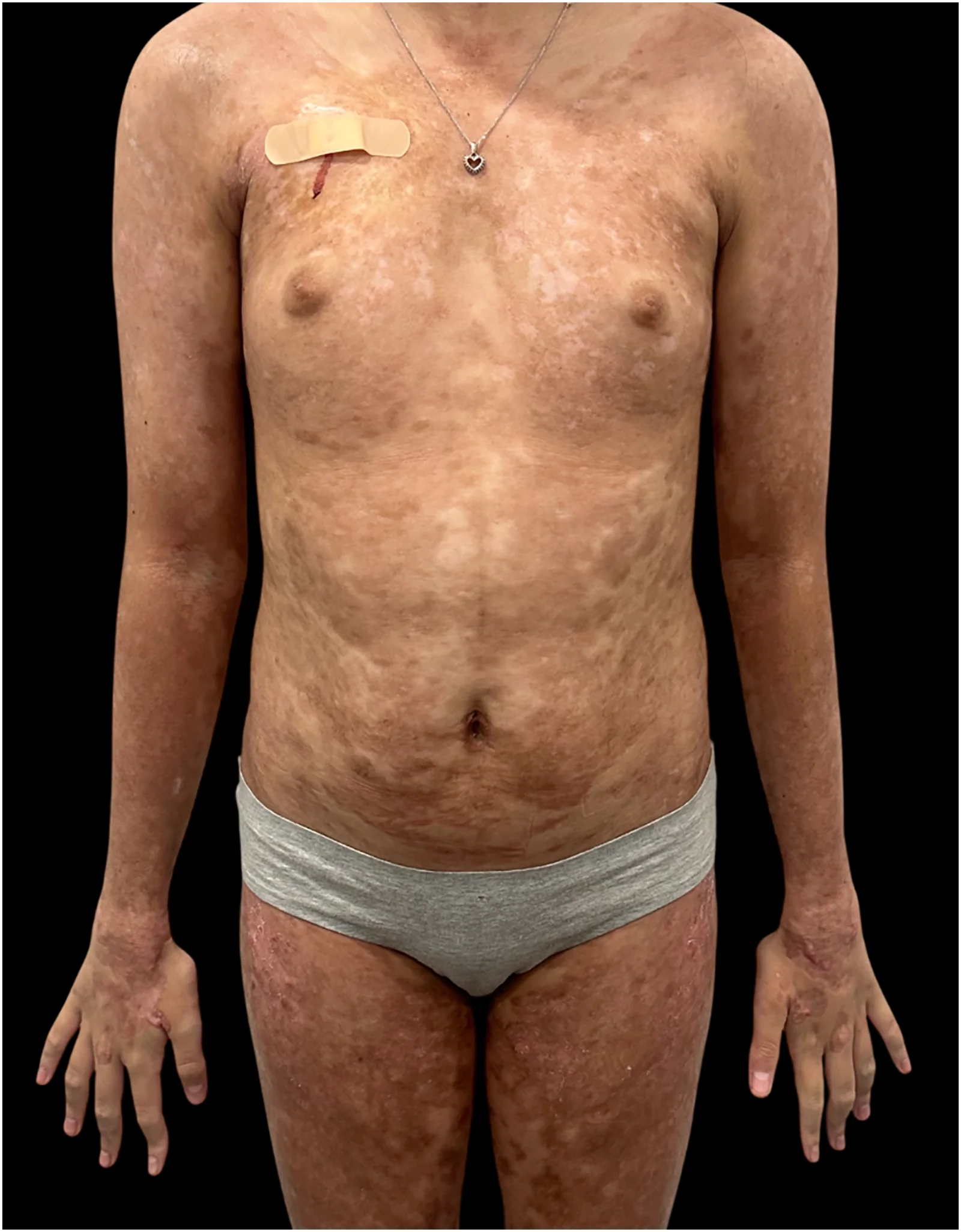
Improvement of diffuse skin sclerosis and atopic dermatitis-like lesions after 10-months of dupilumab treatment, with persistent keloidal plaques on the dorsal hands.

Previous reports have described the use of dupilumab after failure of multiple systemic therapies, including ruxolitinib. However, to the best of our knowledge, concomitant administration of dupilumab and ruxolitinib has not previously been reported in cutaneous cGVHD. In our patient, dupilumab was introduced as an adjunct to ongoing ruxolitinib therapy, and no treatment-related adverse events were observed.^4^ Although evidence regarding this combination remains limited, our case provides additional experience supporting its apparent short-term safety.

Sclerotic and fibrotic manifestations of cGVHD remain among the most challenging forms of the disease to treat, reflecting their complex and incompletely understood pathogenesis.^5^ Although Th2-mediated inflammation has been implicated in atopic dermatitis-like manifestations and may contribute to fibrotic responses, the relative contribution of IL-4/IL-13 signalling to sclerotic cGVHD remains incompletely understood.^2^, ^3^ Our observation suggests that adjunctive dupilumab may have contributed to the improvement of the prominent eczematous manifestations and may have been associated with concomitant clinical improvement in skin induration while the patient remained on ruxolitinib. However, this single observation does not establish a causal role of Th2 signalling in fibrosis.

Interestingly, the paradoxical enlargement of preexisting keloids suggests heterogeneous fibrotic responses to cytokine modulation. Unlike diffuse inflammatory fibrosis in cGVHD, keloids represent localized profibrotic microenvironments with distinct fibroblast populations and signalling pathways.^6^ Variable responses of keloids to dupilumab have previously been reported, underscoring the complexity of IL-4/IL-13 signalling in cutaneous fibrosis.^7^

A limitation of this report is the absence of histopathological confirmation of the sclerotic component, because the biopsy was performed on an eczematous lesion rather than an indurated area. Therefore, the specific contribution of dupilumab to the improvement of skin fibrosis cannot be definitively established. In addition, the concomitant use of ruxolitinib precludes determining the individual contribution of dupilumab to the observed improvement.

To our knowledge, this is the first pediatric report of sclerodermiform cGVHD with associated atopic dermatitis-like features successfully managed with adjunctive dupilumab. Although limited by the single-case nature of the observation, this report suggests that dupilumab may represent a useful adjunctive therapy in selected patients with cGVHD presenting prominent eczematous manifestations, although the contribution to the improvement of the sclerotic component remains uncertain. Further studies are needed to define the efficacy and safety of dupilumab in pediatric cGVHD.

## ORCID ID

Fabià Torres-Betato: 0009-0008-1290-3019

Helena Iznardo: 0000-0002-0710-5521

Ivan López Torija: 0009-0000-9358-6239

Felipe Peirano: 0000-0001-7759-3842

Luís Puig: 0000-0001-6083-0952

Esther Roé: 0000-0003-1601-9227

## Financial support

None declared.

## Authors’ contributions

Fabià Torres-Betato: The study concept and design; data collection, or analysis and interpretation of data; writing of the manuscript or critical review of important intellectual content; critical review of the literature; final approval of the final version of the manuscript.

Helena Iznardo: The study concept and design; writing of the manuscript or critical review of important intellectual content; critical review of the literature; final approval of the final version of the manuscript.

Ivan López Torija: Writing of the manuscript or critical review of important intellectual content; critical review of the literature; final approval of the final version of the manuscript.

Felipe Peirano: Writing of the manuscript or critical review of important intellectual content; critical review of the literature; final approval of the final version of the manuscript.

Luis Puig: Writing of the manuscript or critical review of important intellectual content; final approval of the final version of the manuscript.

Esther Roé: The study concept and design; data collection, or analysis and interpretation of data; writing of the manuscript or critical review of important intellectual content; final approval of the final version of the manuscript; intellectual participation in the propaedeutic and/or therapeutic conduct of the studied cases; effective participation in the research guidance.

## Research data availability

Does not apply.

## Conflicts of interest

Fabià Torres-Betato, Ivan López Torija, and Felipe Peirano have no conflicts of interest to declare. Helena Iznardo has received consultancy/speaker honoraria and/or participated in clinical trials sponsored by AbbVie, Amgen, Boehringer Ingelheim, Novartis, Pfizer, Leo Pharma, Sanofi and UCB. Luis Puig reports receiving research grants/support from AbbVie, Almirall, Amgen, LEO Pharma, Lilly, Sun Pharma, and UCB; and consultancy or speaker’s honoraria from AbbVie, Almirall, Amgen, Biogen, Boehringer Ingelheim, Bristol Myers Squibb, Fresenius Kabi, Horizon (DSMB), J&J Innovative Medicine, LEO Pharma, Lilly, Novartis, Pfizer, Samsung Bioepis, Sandoz, STADA, Sun Pharma, Takeda, and UCB. E.R. has received consultancy/speaker honoraria and/or participated in clinical trials sponsored by AbbVie, Amgen, AstraZeneca, Bayer, Biogen, Cantabria, Celgene, Galderma, Leo Pharma, Lilly, Pierre Fabre, Pfizer and Sanofi.
